# Motivations, consequences, and mechanisms of workplace gossip in nursing groups: a scoping review

**DOI:** 10.3389/fpubh.2025.1672645

**Published:** 2025-12-18

**Authors:** Siying Wei, Yang Yang, Xun Xu, Ling Zhu, Yawen An, Wenjun Hu, Zuoyan Liu, Xiaofeng Xie

**Affiliations:** 1 West China Hospital /West China School of Nursing, Sichuan University, Chengdu, China; 2 School of Mathematics, Southwestern University of Finance and Economics, Chengdu, China; 3 SWUFE-UD Institute of Data Science, Southwestern University of Finance and Economics, Chengdu, China; 4 Lucas College and Graduate School of Business, San José State University, San José, CA, United States; 5 Department of Rehabilitation Medicine, West China Hospital, Sichuan University, Chengdu, China; 6 Innovation Center of Nursing Research and Nursing Key Laboratory of Sichuan Province, West China Hospital, Sichuan University, Chengdu, China; 7 West China School of Nursing, Sichuan University, Chengdu, China

**Keywords:** workplace gossip, nurses, scoping review, motivations, consequences

## Abstract

**Background:**

Workplace gossip is a pervasive form of informal communication, with significant implications for both individual and organizational consequences. Nevertheless, it remains underexplored, particularly within the distinctive social context of nursing profession.

**Methods:**

Using the Arksey and O’Malley framework, a scoping review was conducted across six databases including PubMed and Cochrane Library, retrieving 1,487 articles and examining 30 studies from 1993 to 2025. The selection of studies followed predetermined inclusion and exclusion criteria, and the extracted data were charted using a series of tables.

**Results:**

Grounded in Social Information Processing theory, this study systematically explores the motivations, consequences, and mechanisms of workplace gossip in nursing groups. The findings reveal that: (1) Workplace gossip stems from both intrinsic and extrinsic motivations, deeply shaped by social context of the nursing profession; (2) It has both beneficial and harmful effects on nurses and organizations; (3) Its mechanisms of influence are mediated by organizational and cultural factors such as perceived justice, relational networks, work environment, and group tenure diversity.

**Conclusion:**

As a complex organizational phenomenon, workplace gossip exhibits distinctive motivations, consequences, and underlying mechanisms in nursing groups. A comprehensive understanding and evidence-based guidance of gossip in nursing practice may transform it into a constructive managerial resource, fostering staff well-being, patient safety, and organizational adaptability.

## 1 Introduction

Workplace gossip, defined as “informal and evaluative communication from one organizational member to other members of the same organization about absent others” (1), is an inevitable aspect of social and organizational life (2). As one of the oldest forms of mass communication, workplace gossip is ubiquitous across various organizations and environments (3, 4). Research indicates that gossip constitutes a significant portion of daily workplace interactions (5). Grosser et al. (6) reported that approximately 90% of employees engage in workplace gossip, and Mitra and Gilbert (7) found that it accounts for up to 15% of all work-related emails. In healthcare settings, gossip is similarly prevalent in nursing groups. Empirical studies reveal that 88.1% of nurses acknowledge the presence of gossip in their workplaces (8), with 55.6% reporting frequent exposure and 46% admitting to occasional participation (9).

Historically, workplace gossip has been viewed as unethical behavior with potential social disruptive effects, often associated with negative consequences such as damaged reputations, heightened negative emotions, and decreased work efficiency (10, 11). However, in decades, many studies have begun to highlight the dual nature of workplace gossip, illustrating its potential benefits, including the facilitation of information exchange, emotional expression, and the strengthening of social bonds within organizations (12, 13). This shift in perspective underscores that workplace gossip can have both constructive and destructive impacts, depending on the context and intent (14, 15). In nursing, the impact of workplace gossip is similarly multifaceted. While it can alleviate work-related stress, strengthen social bonds among colleagues, and reinforce organizational culture, it can also provoke anxiety, damage interpersonal relationships, and undermine team cohesion (16). However, unlike in other professional settings, the high-stakes nature of patient care amplifies the potential risks associated with workplace gossip, rendering its negative consequences particularly unpredictable. Any form of gossip may pose significant and long-term threats to patient safety (17). For instance, gossip that distracts staff can impair nurses’ concentration and efficiency, potentially compromising care quality. Moreover, gossip that breaches patient confidentiality can erode trust between nurses and patients, raising serious ethical and legal concerns. Given these risks, it is essential to gain a deeper understanding of workplace gossip within nursing contexts and implement strategies to manage its influence, thereby ensuring a safe, collaborative, and high-quality healthcare environment.

In recent years, workplace gossip has attracted increasing scholarly attention (18, 19). Emerging research highlights its significance in human resource management and individual career development (10, 20). It should be noted that gossip, as a collective social behavior, is profoundly shaped by the social context in which it occurs (2). Therefore, understanding workplace gossip in nursing groups requires situating it within the specific social context of the nursing profession. However, existing research on nurses’ workplace gossip remains limited and fragmented. A comprehensive and integrative framework that captures the motivations, consequences, and underlying mechanisms has yet to be established, thus hindering a coherent understanding of the complete logical chain underlying this prevalent phenomenon among nurses. To address this gap, the study employs Social Information Processing theory to systematically examine the motivations, consequences, and underlying mechanisms of workplace gossip among nurses. According to the Social Information Processing theory, individuals’ attitudes and behaviors are shaped by their perception, interpretation, and active processing of complex informational cues embedded in social contexts (21). Within the nursing profession-characterized by high work pressure, intensive interpersonal interaction, and significant emotional labor-workplace gossip can be viewed as a behavioral manifestation of nurses’ active perception, interpretation, and response to social information cues when confronted with the multifaceted informational and emotional demands of their work (22). In this study, social information cues are defined as the various forms of social information that nurses encounter and interpret in their daily work-such as high-pressure, information-intensive work environments, leadership styles, adjustments in workload or scheduling, and patients’ individualized care needs. Through the perception and interpretation of these cues, nurses develop psychological and social needs at the individual level, including the need for information acquisition, emotional regulation, and social interaction. These needs may promote gossip behavior, ultimately leading to diverse consequences at both individual and organizational levels. From this theoretical perspective, this study identifies the intrinsic and extrinsic motivations underlying nurses’ engagement in gossip, elucidates its behavioral pathways and influencing factors, and interprets its positive and negative consequences. These findings provide a theoretical foundation for advancing research on workplace gossip in nursing groups and offer practical implications for guiding organizational management and fostering healthier, more collaborative, and adaptive work environments.

## 2 Methods

This scoping review followed the PRISMA extension for scoping reviews (PRISMA-ScR) guidelines (23), which consists of five key steps: identifying research questions, searching for relevant studies, study selection, charting the data, and collating, summarizing, and reporting the results.

### 2.1 Identifying research questions

This review was designed to answer the following questions: (1) What motivates nurses to engage in workplace gossip within the unique professional context of nursing? (2) What are the consequences of workplace gossip? (3) How does workplace gossip exert its influence on nurses?

### 2.2 Searching for relevant studies

An extensive literature review was conducted to identify key terms related to gossip. The following search terms were used: “gossip,” “workplace gossip,” “whispers,” “rumors,” “informal communication,” “informal knowledge transfer,” “informal knowledge sharing,” “individual behavior,” and “grapevine.” Six electronic databases were searched: PubMed, Embase, Medline, Cochrane Library, Web of Science, and CINAHL. The search cutoff date was March 26, 2025. To ensure comprehensive coverage of relevant literature, truncation symbols (∗) and Boolean operators (“OR” and “AND”) were used, along with various combinations of subject headings, keywords, and other search terms (24). No restrictions were applied regarding publication date or article type. Additionally, the reference lists of retrieved articles were manually examined to identify other relevant studies. The search strategy of PubMed detailed in the Supplementary material.

### 2.3 Study selecting

Following the Preferred Reporting Items for Systematic Reviews and Meta-Analyses (PRISMA) guidelines, all authors collaboratively discussed and established the final inclusion and exclusion criteria (25). Studies focused on workplace gossip among nurses were included. Given the limited literature on workplace gossip within nursing contexts, studies involving other healthcare professionals, as well as those lacking empirical data (e.g., reviews, commentaries), were also included. The exclusion criteria included publications not in English and Chinese, studies focused on informal nursing roles (e.g., interns or undergraduate nursing students), and papers without an abstract or those inaccessible in full text.

**Figure 1:**
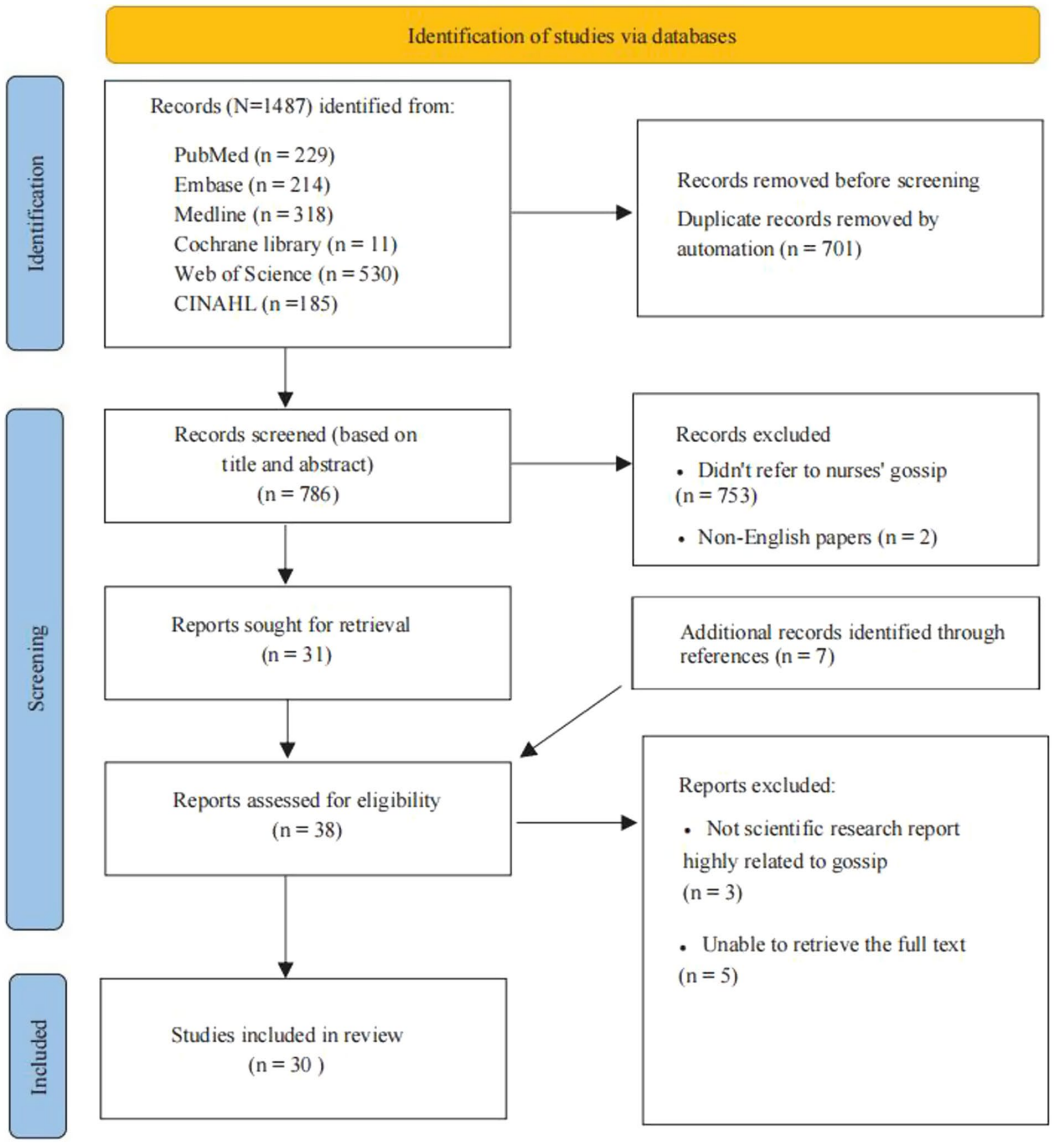
PRISMA flow diagram.

To ensure compliance with the criteria, two authors independently screened the articles following the established procedures. Discrepancies were resolved through discussion to reach a final consensus. The screening process involved three stages: removal of duplicates, initial screening (reading titles and abstracts), and full-text review. A total of 1,487 studies were identified from the initial database search. The search results were imported into EndNote bibliographic software, where duplicate records were removed. Further screening was conducted based on study titles and abstracts, followed by manual searching of reference lists from the included studies to identify additional relevant literature. After rigorous examination, 30 studies were deemed eligible for full-text inclusion. A detailed flowchart of the study selection process is presented in Figure 1.

### 2.4 Data charting

Data were extracted independently by two authors. Any discrepancies were resolved by consensus, with a third author consulted when needed for further clarification (26). Data extraction followed the JBI guidelines for scoping reviews, including author(s), year, country, objectives, study design, participants and their locations, main findings, and limitations (27).

### 2.5 Collating, summarizing, and reporting the results

Data analysis followed a general inductive approach. Initial categories were developed through discussion, and the extracted data were subsequently examined and consolidated. Results were synthesized by summarizing key study details and findings. Study characteristics were tabulated for clarity, as shown in Supplementary Table 1.

## 3 Results

### 3.1 Description of the studies

Supplementary Table 1 presents the characteristics of the 30 studies included in this review, comprising 27 peer-reviewed articles, 2 commentary articles (28, 90) and one doctoral dissertation (29). All publications were in English. In these studies, the most common research design was cross-sectional, used by nine studies (8, 9, 16, 30–35). Five studies employed qualitative designs (17, 36–39), three used mixed methods combining quantitative and qualitative approaches (29, 40, 88), and one was a longitudinal design (41). The remaining 12 studies were non-empirical, primarily literature reviews. Regarding publication year, only eight studies were published within the last 5 years (2021–2025). Among the 18 empirical studies, most were conducted in Turkey (*n* = 7, 38.89%), followed by the United Kingdom (*n* = 3, 16.67%), South Korea (*n* = 2, 11.11%), and Egypt (*n* = 2, 11.11%). One study was conducted in each of the following countries: Iran (*n* = 1, 5.55%), Germany (*n* = 1, 5.55%), Canada (*n* = 1, 5.55%), and other European countries (*n* = 1, 5.55%). The majority of the included studies (*n* = 24, 80%) exclusively investigated nurse populations, while the remaining six studies employed mixed samples consisting of nurses and other healthcare professionals. Among these, three were empirical studies in which nurses constituted no less than 31.1% of the participants (16, 33, 38), two of these studies reported subgroup analyses specifically for nurses (33, 38). In addition, the other three non-empirical studies provided narrative reviews addressing gossip phenomena within the broader healthcare professionals (42–44). The studies were conducted in various healthcare settings, including national or university hospitals and nursing homes. Regarding the clinical settings, one study was conducted in pediatric units (30), another in inpatient wards (9), and two studies were undertaken in internal medicine or surgery clinics (37) and across multiple older adult care homes (39). The remaining studies did not specify the departments of the nursing participants.

In the quantitative studies, several widely used scales from other fields were applied. Specifically, The Gossip and Rumor Attitude Scale developed by Eşkin Bacaksız (45) and the scale developed by Wittek (46) were each used in three studies. Two studies employed the Gossip Functions Questionnaire proposed by Foster (47), while one study utilized a Supervisor Positive Gossip Scale, like the three-item scale by Ugwu et al. (48). However, fewer than half of these studies assessed the validity and reliability of the scales used.

### 3.2 The definition of workplace gossip

Workplace Gossip was firstly conceptually highlighted in organizational life by Noon and Delbridge (49). Given its foundation in various theoretical perspectives, it has since been conceptualized in multiple ways (50, 51). Among the 30 studies on workplace gossip in nursing, over half (*n* = 16, 53.33%) did not provide an explicit definition of workplace gossip. In contrast, the definitions of workplace gossip varied across the remaining 14 studies (see Table 1). Of these, 13 studies included the valence (i.e., the positive or negative nature) in their definitions, while (43) excluded the valence from their definition.

**Table 1:** Definition of workplace gossip.

Author, year	Definition of workplace gossip	Including valence
Özlük and Özcan (9)	An informal form of communication, often involving conversation aimed at backbiting or condemning others.	Yes
Şantaş (16)	Informal, evaluative communication about an absent member of an organization, involving the exchange of value-laden information.	Yes
Ceylan and Çetinkaya (30)	A spontaneous form of verbal communication that two or more people use to praise or denigrate people, and to share valuable and important information.	Yes
Georganta, et al. (33)	An informal, evaluative communication among a few individuals, about another who is or is not present.	Yes
Zoromba, et al. (35)	Supervisor positive gossip is defined as informal, favorable talk among superiors and team members regarding a colleague from the organization who is not present.	Yes
Kim, et al. (34)	Informal and evaluative (i.e., positive or negative) talk from one member of an organization to one or more members of the same organization about another member of the organization who is not present to hear what is said.	Yes
Kim, et al. (41)	Informal and evaluative communication from one organizational member to other members of the same organization about absent others.	Yes
Altuntaş (38)	A spontaneous form of verbal communication that two or more people use to praise or accuse people and to share valuable and important information.	Yes
Altuntaş, et al. (37)	A communication style widely used by at least two people or by small, mostly unplanned, groups to praise and blame people, and to share valuable or important information.	Yes
Begemann, et al. (39)	Informal and evaluative (i.e., positive or negative) talk from one member of an organization to one or more members of the same organization about another member of the organization who is not present to hear what is said.	Yes
Begemann, et al. (39)	An evaluative informal discussion about the social environment member who is absent.	Yes
Aghbolagh, et al. (36)	The exchange of evaluative information within informal communication networks, involving two or more individuals, with the target not present.	Yes
Waddington (44)	Informal, private communication between an individual and a select audience about the behavior of absent individuals or events, often involving implicit or unstated evaluations.	Yes
Montgomery, et al. (43)	The process of informal communication about individuals within a social setting, involving at least two people.	No

**Figure 2:**
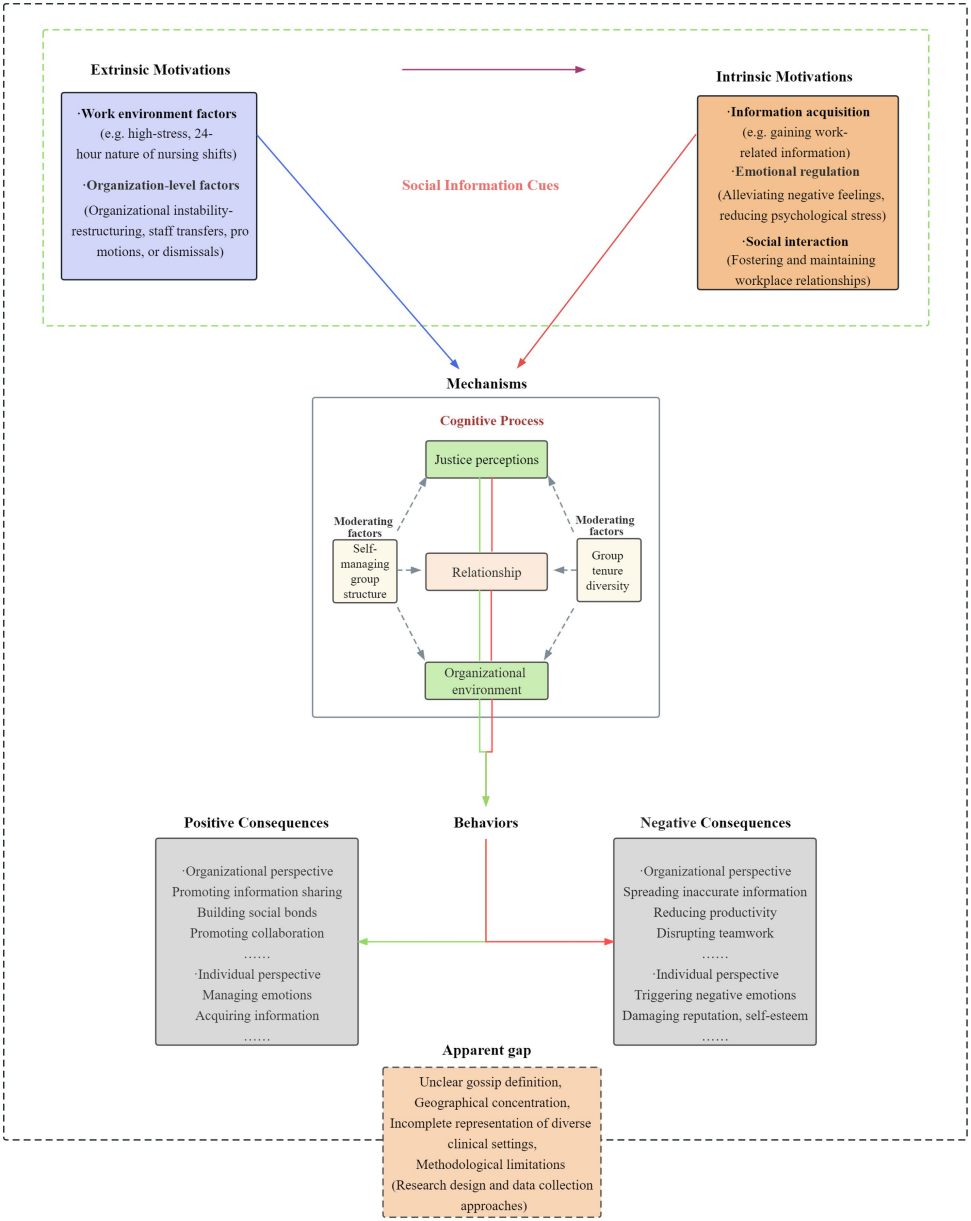
Theoretical framework of workplace gossip behavior in nurses groups.

The following sections explore the motivations, consequences, and mechanisms of workplace gossip in nursing groups. To enhance understanding of these interconnected aspects, this study adopts Social Information Processing theory as its guiding theoretical framework. Within this framework, workplace gossip among nurses is conceptualized as an adaptive response to complex social information cues embedded in the nursing context (21). Specifically, through the active perception and subjective interpretation of diverse social information cues in their work environment - including the inherent occupational characteristics of high work pressure, intensive interpersonal interaction, and substantial emotional labor (8, 30, 36, 43), as well as dynamic organizational events such as changes in leadership style (8, 36, 38), adjustments in workload or scheduling, fluctuations in patients’ clinical conditions, and potential exposure to workplace aggression or incivility (52, 53)—nurses develop varied psychological and social needs, such as information acquisition or emotional regulation. These needs trigger gossip behavior, which subsequently exerts both positive and negative influences at individual and organizational levels. Based on this integrative theoretical perspective, Figure 2 presents the Theoretical Framework of Workplace Gossip Behavior in Nurses Groups, offering a systematic synthesis and theoretical representation of its motivations, consequences, and underlying mechanisms.

### 3.3 The motivations of workplace gossip

From the perspective of Social Information Processing theory, workplace gossip among nurses is driven by diverse social information cues originating from both the work environment and organizational level (22). These cues subsequently trigger individual-level needs, which collectively promote the emergence of gossip behavior. Accordingly, we propose that the motivations for gossip among nurses can be categorized into two primary types: extrinsic and intrinsic motivations. These categories are further illustrated in Table 2.

**Table 2:** The motivations of workplace gossip.

Motivations	Domain	Findings	Source(s)
Extrinsic motivations	Work environment factors (13)	High-stress and fast-paced work environments contribute to an increased volume of information exchange.	(8, 16, 30, 36, 43, 55)
		The 24-h context of nursing work, and the demands of collaborating with many colleagues within and across teams expose nurses to a considerable amount of information related to day-to-day care.	(17, 32, 39, 43, 54)
		Exclusive reliance on formal information-sharing channels fails to meet the communication needs of nurses, to provide high-quality care, effective communication need occur through both formal and informal channels.	(8, 32, 38, 43)
		Nurses provide care to individuals under significant stress while also contending with greater emotional pressures themselves.	(8, 9, 30)
	Organization-level factors (3)	When organizational issues arise, nurses are more likely to engage in gossip, such as when tasks are unclear, structural flaws exist, formal communication channels are ineffective, or during crises and periods of uncertainty.	(8, 36, 38)
Intrinsic motivations	Information acquisition (11)	The need to gain work-related information to achieve high-quality care.	(8, 9, 17, 32, 36–39, 56, 57)
		The need to obtain or share information of interest, such as financial matters, individual merits, and job conditions.	(36)
	Emotional regulation (11)	Gossip can offer relief from emotionally charged situations and serve as a means of reassurance and support.	(8, 9, 17, 30, 36–38, 40, 54, 56, 65)
	Social interaction (6)	A way to share information about social events in the personal and professional, strengthening bonds through shared values, common interests, and professional camaraderie.	(38, 40, 43, 55, 57, 59)

#### 3.3.1 Extrinsic motivations

Extrinsic motivations are driven by factors related to the work environment and organizational conditions which create a context where informal communication, such as workplace gossip, is more likely to thrive.

First, we argue that environmental factors are more likely to trigger external motivations. As numerous studies have indicated, hospitals—characterized by high-intensity environments, high patient volume and multifaceted personnel interactions—significantly increase the frequency of gossip (16, 30, 54, 55). Nurses, working in these high-pressure environments (36), navigate complex relationships with both patients and colleagues (8). Given the 24-h nature of nursing shifts and the continuous healthcare demands of patients (39), nurses are frequently exposed to and required to share substantial amounts of information regarding daily care, incidents, and concerns (43). In such a information-saturated environment, workplace gossip often emerges as an informal yet crucial means of communication.

Second, employees are more likely to engage in workplace gossip in response to organizational challenges (38, 43). When organizational tasks are poorly defined or structural issues, such as ineffective leadership or management practices arise, workplace gossip becomes more prevalent. Similarly, during periods of organizational instability—including restructuring, staff transfers, promotions, or dismissals—the frequency of workplace gossip tends to increase as a means of gathering and disseminating information. In summary, these organizational factors create an environment where workplace gossip serves as both a coping mechanism and an essential communication tool for nurses.

#### 3.3.2 Intrinsic motivations

Through the interpretation and processing of social information cues derived from the work environment and organizational context, workplace gossip tends to be formed and disseminated by nurses as a means of fulfilling various personal needs. A review of the existing literature identifies three key intrinsic motivations for workplace gossip among nurses: information acquisition; emotional regulation and social interaction.

The first aspect that may trigger workplace gossip among nurses, as highlighted in numerous studies, is information acquisition (8, 9, 17, 36, 37, 39, 43, 56, 57). Begemann et al. (39) found that, compared to other organizations, healthcare systems have more complex structures and communication methods. As a key conduit of information within healthcare institutions, nurses spend a significant portion of their work interacting and communicating with others. However, formal communication often fails to meet nurses’ needs. Informal channels, such as workplace gossip, help bridge this gap by providing essential work-related information and supplementing details not fully conveyed through formal channels (17). These informal interactions thus play a crucial role in ensuring smooth workflows and informed decision-making in daily tasks. Besides, in organizations, members experience both competitive and collaborative dynamics. Like other professionals, nurses need information to support both personal and professional development (56). When formal communication in organizations is incomplete or unclear, nurses may resort to informal networks out of curiosity or a need to address informational gaps (17). In some cases, they may even unconsciously fabricate details to fill informational gaps, which can contribute to the spread of workplace gossip.

The second factor contributing to the intrinsic motivations for workplace gossip is emotional regulation, as identified in previous studies. Research has shown that the tendency to engage in workplace gossip is significantly associated with occupational preferences (17). Nurses, who are often considered emotional laborers in high-pressure environments, are required to care for patients under considerable stress (8, 9, 17, 36, 37, 39, 43, 56, 57). They frequently encounter intense negative emotions such as pain, suffering, and aggression, while simultaneously being expected to respond with empathy, kindness, and hope (17). This emotional burden is further exacerbated by frequent experiences of bullying and incivility from colleagues, patients, or families—challenges that nurses face globally (52, 53). However, due to their unique role and noble mission, nurses are often expected to suppress these negative emotions and maintain rationality and responsibility to ensure smooth care delivery (58). In this context, workplace gossip provides a crucial emotional outlet. By sharing their experiences and emotions, nurses can alleviate negative feelings and reduce psychological stress. The consistent findings in the literature suggest that workplace gossip plays a crucial role in helping nurses manage work-related stress, regulate emotions, and maintain mental well-being (8, 9, 17, 36, 37, 39, 43, 56, 57).

The last aspect related to intrinsic motivations is social interaction. As a high-intensity and interactive profession, nurses face challenges not only in their work relationships with patients but also in balancing multiple roles and responsibilities in their personal lives (38). In fact, workplace gossip among nurses often extends beyond professional concerns to include personal matters, such as family responsibilities, stress, and aspects of their social lives (9). Through sharing similar experiences and discussing personal issues with colleagues, nurses seek advice and reassurance from others who face similar challenges (40, 43, 44, 57, 59). In this sense, interactions through workplace gossip create a supportive network, enhancing emotional resilience and promoting a sense of camaraderie in a profession often marked by high emotional demands.

### 3.4 The consequences of workplace gossip

Based on Social Information Processing theory, nurses engage in a series of active cognitive and emotional processes while perceiving and interpreting social information cues from multiple sources in the workplace. These differential information processing approaches may lead to divergent consequences. It should be noted that within the high-pressure, highly interactive, and emotionally demanding context of the nursing profession (8, 30, 36, 43), the consequences of workplace gossip exhibit distinctive characteristics: its disruptive effects are intensified by the high-risk nature of nursing, while its potential benefits are amplified by the interdependence within the team, as shown in Table 3.

**Table 3:** Consequences of workplace gossip.

Consequences	Domain	Findings	Source(s)
Positive consequence	Organizational perspective (10)	Workplace gossip can promote communication and information sharing.	(17, 28, 37, 38, 43, 55, 57, 63)
		Workplace gossip builds social bonds by developing or reinforcing common interests and comradeship within the nursing unit.	(28, 43, 55, 57, 63)
		Workplace gossip promotes collaboration and produces more effective teamwork and efficiencies.	(17, 55, 57)
		Workplace gossip can establish and maintain work-related group norms and values.	(28, 39, 55, 57, 63)
		Workplace gossip informs management about organizational trends, prevailing views on current issues, the general concerns, and productivity of employees can facilitate effective organizational management.	(63)
		Workplace gossip is a valuable early warning indicator of risk and failure.	(43, 44)
	Individual perspective (18)	Workplace gossip is an essential way to acquire information.	(36, 37, 56)
		Workplace gossip helps nurses manage negative emotions such as stress and worry.	(8, 17, 29, 36–40, 43, 54–57, 63, 88)
		3. Workplace gossip meets nurses’ social needs.	(17)
		4. Workplace gossip serves a self-evaluative function: evaluating nurses’ behavior against workplace norms.	(63, 88)
		5. Workplace gossip promotes the professional socialization of nurses.	(28, 31)
		6. Supervisor positive gossip can weaken the negative effect of mistreatment by patients on nurses, such as psychological detachment and caring behaviors.	(35)
		7. Workplace gossip helps managers make reasonable decisions.	(36)
Negative consequences	Organizational perspective (8)	Workplace gossip may convey inaccurate or incorrect information that can lead to mistakes and fatal errors.	(8, 17, 33, 55, 63)
		Negative workplace gossip reduces productivity, disrupts teamwork, damaging nursing quality and patient safety.	(16, 17, 54, 55, 57)
		Negative workplace gossip even leads to a negative image of the unit or even the entire hospital facility.	(55)
	Individual perspective (15)	Workplace gossip leads to negative emotions—guilt, stress, pain, tension—impacting well-being.	(8, 16, 33, 38, 40, 57, 65, 90)
		Negative workplace gossip damages nurses’ reputation.	(8, 17, 29, 39, 54, 55)
		Negative workplace gossip undermines targets’ self-esteem.	(29)
		Negative workplace gossip erodes targets’ self-confidence.	(65)
		Negative workplace gossip leads to lower job satisfaction, higher occupational burnout.	(90)
		Workplace gossip negatively correlates with nurses’ engagement, care quality.	(33, 44)
		Negative gossip breaches patient privacy, undermines trust.	(30)

#### 3.4.1 The negative consequences of workplace gossip

Workplace gossip has long been viewed primarily as a negative phenomenon, particularly in high-stakes settings like healthcare, where its consequences can be profoundly harmful.

Workplace gossip is not a static process; rather, it is dynamic and multifaceted, involving three key parties: the initiator (who actively spreads gossip), the recipient (who overhears or passively participates), and the target (the person being discussed) (60, 61). In organizational settings, workplace gossip can shift fluidly among colleagues, with any individual potentially becoming the target. When gossip becomes malicious or unchecked, it can affect all involved parties. For the target, the negative consequences of workplace gossip are particularly severe. Studies have linked mocking or derogatory gossip to workplace bullying, which can severely damage the target’s emotional health (29). Nurses targeted by malicious gossip may experience emotional exhaustion, depersonalization (33), and a loss of self-esteem and reputation (28). In severe cases, this can lead to reduced work engagement, higher burnout risk, and a decline in care quality (54, 57). For the initiator, spreading negative gossip can lead to feelings of guilt and psychological distress. As noted by Altuntaş (38), nurses often experience emotional burdens and regret after engaging in malicious gossip. Moreover, negative gossip can conflict with workplace norms and ethics, with the initiator potentially facing accusations of making unverified attacks on colleagues (38). This can damage the initiator’s credibility and undermine relationships and trust within the team. For recipients, prolonged exposure to negative gossip can undermine psychological safety (55, 62, 63). While occasional exposure may encourage self-reflection, continuous exposure can foster fear of becoming the next target, leading to social withdrawal, avoidance of close relationships, and reluctance to express opinions.

At a broader level, workplace gossip extends beyond individual interactions, significantly influencing overall organizational dynamics and the work environment. It can heighten interpersonal tensions, exacerbate trust issues among nurses, and undermine teamwork and collaboration, ultimately fostering a toxic atmosphere. This pervasive negativity can directly compromise patient safety, as reduced cooperation among nursing staff increases the risk of errors and poor patient outcomes (16, 17, 54, 55, 57, 63). Furthermore, as gossip spreads, it can damage the organization’s professional reputation (55).

#### 3.4.2 The positive consequence of workplace gossip

While workplace gossip is often perceived negatively and linked to unfavorable outcomes, recent studies indicate that, when effectively managed, it can serve as a valuable organizational resource, benefiting both organizations and individuals.

From an organizational perspective, workplace gossip can positively contribute to various organizational dynamics (33, 36–39, 42–44, 55, 56, 59). Workplace gossip in healthcare institutions helps facilitate the dissemination of information, filling gaps left by formal communication channels. This fosters a more dynamic exchange of information, bridging the communication divide and enhancing organizational transparency (37). Furthermore, through workplace gossip, nurses share personal matters and exchange viewpoints, thereby fostering close relationships and enhancing teamwork (55, 57). Moreover, workplace gossip plays a crucial role in shaping and maintaining organizational culture by conveying unwritten rules and values that govern behavior (64). It is particularly influential during times of uncertainty and organizational change (33). In such circumstances, workplace gossip acts as an informal social control mechanism, reinforcing organizational norms and expectations. Both praise-based and judgmental gossip subtly influence employee behavior, encouraging alignment with organizational norms. This process contributes to the stability and long-term development of the organization. Finally, as a potential resource for human resource management, workplace gossip can offer valuable insights into employees’ perceptions of their work environment and highlight underlying organizational issues (43, 44, 63). It allows managers to identify employee complaints and suggestions early, enabling timely intervention before significant problems emerge. For example, when a new policy is introduced, managers can assess employee reactions through workplace gossip. If the gossip mainly reflects dissatisfaction, it may indicate potential issues with the policy, providing an opportunity for managers to address these concerns (43). In healthcare settings, where even minor errors can have major consequences for patient safety, workplace gossip can serve as an early warning system for potential dysfunctions within the organization. It helps managers identify emerging problems that could pose substantial risks, offering valuable insights into professional practice and patient safety (44).

From individual perspective, workplace gossip can alleviate stress among nurses and provide emotional support. First, workplace gossip offers nurses a temporary escape from the emotional challenges encountered in their interactions with patients and colleagues. This temporary mental disengagement helps maintain emotional balance, alleviates the accumulation of negative emotions, and reduces their impact on both mental health and job performance (8, 29, 37–40, 43, 54). Second, Zoromba et al. (35) found that positive workplace gossip from nursing leaders can enhance team resilience, particularly in the face of professional challenges such as patient abuse. It has been shown that positive workplace gossip helps reduce feelings of psychological detachment and lowers the risk of burnout, which ultimately induce greater work engagement and higher-quality nursing care. In addition, many studies have mentioned the influence of workplace gossip in facilitating professional socialization of nurses. Several scholars, such as Thomas and Rozell (55), Ribeiro and Blakeley (59), and Baltimore (65), have emphasized that experienced nurses can use workplace gossip to transmit organizational rules, behavioral norms, and values to newly hired nurses. Through sharing personal experiences and insights, they provide essential support and guidance, helping new nurses develop a sense of belonging, strengthen their professional identity, and make them quickly adapt to work environment and integrate into the team.

### 3.5 The mechanisms of workplace gossip

According to Social Information Processing theory, nurses’ perception and processing of social cues constitute a dynamic process that is influenced by the broader social context. As a key feature of this context (66), organizational culture plays a critical role in shaping this process, moderating the relationship between gossip behavior and its consequences. Existing studies have sporadically identified several pathways through which workplace gossip exerts its influence (16, 34, 35, 38, 41, 55), as summarized in Table 4. Through systematic synthesis of existing studies, we found that these mechanisms are not isolated but are embedded within the broader context of organizational culture.

**Table 4:** The mechanisms of workplace gossip.

Mechanism type	Domain	Mechanism description	Source(s)
Main mechanism pathways	Justice perceptions	Gossip’s biased evaluations can distort perceptions of justice, influencing behavioral responses.	(34)
	Relationship	Workplace gossip may yield positive or negative consequences by facilitating or changing the relationship between parties.	(35)
	Organizational environment	Workplace gossip can reshape the organizational environment nurses face, subsequently impacting their performance.	(16, 38, 55)
Moderating factors	Group tenure diversity	Increased tenure diversity restricts the negative functioning of workplace gossip, thereby reducing its adverse effects.	(41)
	Self-managing group structure	Stronger self-managing structures within nursing teams enhance the positive mechanisms of workplace gossip, facilitating information processing and promoting positive behavioral outcomes.	(41)

Drawing on Social Information Processing theory, humans rely on cultural tools to interpret and make sense of their world (66). Within this framework, workplace gossip can be conceptualized as an informal informational tool that enables nurses to acquire and construct perceptions of organizational justice, which promotes the emergence of either positive or negative consequences. Perceived justice refers to employees’ justice perceptions of the treatment they have received (67). It emanates from employees’ subjective evaluations of their experience in organizations (68, 69). During the intensive communication process of workplace gossip, it can transmit a non-objective and biased evaluation of the target and distort the recipient’s perceived justice which consequently leads to positive or negative feedback from the recipient (70). Moreover, justice perceptions influence the spread of positive or negative workplace gossip (6). Therefore, nurses’ perceived justice partially emanates from the information initiated by workplace gossip, which in turn represents their immediate behavioral response. Based on social exchange theory, Kim et al. (34) have discussed the interaction between workplace gossip and four types of justice perceptions among 329 nurses. Their conclusions proved that the interaction between workplace gossip and perceived justice plays an important role in the mechanism of workplace gossip.

From the emotional perspective of organizational culture, the relationship among participants serves as an alternative mechanism through which workplace gossip exerts its effects. Workplace gossip is a critical social conversation about individual and specific behaviors in a sense (71). Such informal conversations, which are outside the hierarchical structure of organization, often acts as a vital tool for reinforcing relationship between the parties involved. Thus, some scholars have pointed out that the workplace gossip may yield positive or negative consequences by facilitating or changing the relationship between parties involved (72). For example, Grosser et al. (89) have found that when two gossipers in workplace gossip have a close friendship, the influence of workplace gossip, either positive or negative, may be amplified. Besides, Zoromba et al. (35) have focused specially on the workplace gossip between nurses and their supervisors, revealing that positive gossip from supervisors reduced nurses’ psychological detachment attributable to adverse workplace factors.

Additionally, building on existing studies, gossip can be understood as both an expression of organizational culture (8), offering valuable insights into work quality and the organizational climate (33), and a cultural practice that actively contributes to the ongoing reconstruction of the organizational environment. Studies have proved that workplace gossip may increase or decrease the environmental pressure faced by nurses, leading to either positive or negative consequences (16, 38, 55). This conclusion aligns with findings by L’Huillier et al. (73). They found that surgery residents viewed gossip as social capital, using it to influence their power within the organization and acquire social resources, thereby shaping behavior. Also, workplace gossip can lead to adverse effects, such as undermining nurses relationships by dressing up a toxic structure and harm the organizational climate (16). Thus, it may exert positive or negative impacts by influencing the organizational environment.

Finally, the mechanisms of workplace gossip may be moderated by organizational characteristics such as group tenure diversity and self-managing structures. Group tenure diversity refers to the variation in organizational tenure among nursing team members, which reflects differences in job-related attributes, experience, and expertise (34). It impacts team interactions by enhancing explicit knowledge (74) and diversifying perspectives (75), thus mitigating negative gossip effects (76). Kim et al. (41) found that high tenure diversity reduces gossip’s negative impact by broadening task-related discussions. Moreover, as shown by Kim et al. (41), self-managing structures strengthen interdependence and decision-making efficiency, positively moderating gossip’s role. These structures enhance information exchange and foster positive consequences in nursing teams.

## 4 Discussion

This scoping review aimed to explore workplace gossip among nurses.

First, workplace gossip is difficult to accurately define (77, 91). The definition by Brady et al. (1), which characterizes gossip as “evaluative (positive or negative)” or “valence-based” communication, is widely used. However, recent studies suggest that gossip can also be non-evaluative and mainly serves as an information exchange (43, 78). In this regard, Dores Cruz et al. (77) proposed a broader definition, describing workplace gossip as “a sender communicating to a receiver about a target who is absent or unaware of the content.” This challenges the prevailing assumption that workplace gossip is always evaluative. In our review, nearly half of the studies do not define workplace gossip clearly, and those that do show significant variation. Most studies still define it as an evaluative form of communication. However, an observational study has revealed that non-evaluative, neutral-valence gossip is common among nurses (39). Based on these findings, we suggest adopting the definition proposed by Dores Cruz et al. (77) to standardize the understanding of workplace gossip within nurses.

Second, as emphasized by Fan and Grey, (2), “gossip is a highly socialized behavior,” the meaning and manifestation of which are profoundly shaped by specific social and cultural contexts. Detached from its contextual background, the essence and functions of gossip cannot be fully understood. A systematic review of the extant literature suggests that empirical evidence concerning workplace gossip among nurses is still insufficient. One primary limitation pertains to the scarcity of empirical research. Among the 30 studies included in the review, only 18 were empirical studies, spanning a period of 31 years (1993–2025), which reflects the limited scholarly attention devoted to the topic of gossip among nurses. The paucity of research may be largely attributable to the inherently private and culturally sensitive nature of gossip (30), as well as to the high moral sensitivity and stringent ethical norms that characterize the nursing profession (17). These factors impose substantial ethical constraints on researchers during study design and implementation, thereby limiting both the scope and feasibility of related empirical investigations. A further limitation relates to the marked geographical concentration of empirical investigations, nearly half of which have been conducted in Turkey, while research originating from East Asia remains notably scarce. This imbalance may be attributed to regional variations in sociocultural factors. Specifically, as a transcontinental nation spanning Europe and Asia, Turkey exhibits a unique blend of Islamic and European cultural characteristics (79). Its social structure and value system are characterized by a distinct “high collectivism–high power distance” configuration (80). The collectivistic orientation reinforces close interpersonal networks within groups and facilitates informal information exchange, whereas the hierarchical organizational culture constrains formal communication channels, making gossip an important medium for emotional expression and information dissemination. Moreover, the highly feminized nature of the nursing profession, together with deeply ingrained traditional gender role expectations in Turkish society (81), further amplifies nurses’ psychological need to engage in gossip as a means of emotional regulation and identity negotiation, rendering this group a representative sample for studying such behaviors. In contrast, research progress in East Asia appears to be constrained by distinct sociocultural factors. Although countries such as Japan and China have large nursing workforces, the Confucian emphasis on “harmony” and “face” creates substantial cultural and institutional barriers to the open discussion of workplace gossip (82, 83). These deeply embedded sociocultural norms have consequently limited the development of empirical research on this topic. It is therefore suggested that the current geographical concentration of studies on nurses’ workplace gossip is closely associated with specific cultural factors. Most notably, a systematic review and synthesis of existing studies on workplace gossip among nurses reveal considerable heterogeneity in research designs and focal emphases. Most studies have paid insufficient attention to macro-level determinants, particularly sociocultural contexts, thereby impeding a systematic understanding of cultural variations in nurses’ workplace gossip and constraining the validity of cross-cultural comparisons. Moreover, the only cross-national investigation identified to date reported no significant differences in nurses’ gossip behaviors (33), further highlighting the urgent need for comprehensive and methodologically rigorous comparative research across diverse cultural settings.

Further, previous studies have predominantly focused on clinical settings like inpatient wards and older adult care (9, 30, 37, 39), overlooking dynamic environments like outpatient clinics and ICUs, where factors like high patient turnover and complex information flows significantly influence gossip (84, 85). Therefore, cross-sector comparisons of workplace gossip across different workplace settings are still needed. Additionally, regarding study populations, six studies that primarily sampled nurses while also including other healthcare professionals were incorporated into this review. Of these studies, three were empirical investigations, two of which provided separate analyses for nurse subgroups. One study identified a high degree of consistency across professional groups, indicating that negative workplace gossip exerts similarly adverse effects on emotional exhaustion, depersonalization, and patient safety (33). In contrast, another study revealed significant differences among professional groups in the motivations for gossip, emotional responses, and coping strategies in response to others’ gossip (38). Specifically, nursing professionals tended to view gossip as a means of emotional relief and social bonding, whereas dental and medical professionals more often engaged in gossip for purposes of criticism or evaluation. In terms of emotional responses, nurses reported the highest levels of post-gossip guilt and a stronger tendency to avoid future gossip. In contrast, physicians primarily experienced stress relief or an urge to verify information, with minimal moral discomfort. When addressing gossip initiated by others, nurses were more likely to employ active intervention strategies, such as attempting to dissuade or stop gossip about colleagues, while physicians predominantly opted for silence or passive listening. These findings indicate that even within similar healthcare organizational contexts, gossip behaviors demonstrate significant professional variations. Nurses tend to display more affective, relationship-oriented, and morally constrained characteristics compared with other medical professionals. Additionally, the remaining four studies that did not provide nurse-specific subgroup data extended the research perspective to broader healthcare settings. These investigations similarly revealed the pervasiveness of gossip within medical systems and its potential implications, with their primary conclusions being largely consistent with findings derived specifically from nursing populations.

Third, the informal and confidential nature of workplace gossip poses methodological challenges in empirical research (5). Nearly half of studies rely on literature reviews and theoretical discussions, with few empirical analyses predominantly using cross-sectional surveys; longitudinal, observational, and intervention-based studies are scarce. Moreover, most cross-sectional studies use questionnaire surveys, but questionnaires adapted from other fields often lack validated measures specific to nursing contexts, compromising their applicability.

Finally, grounded in Social Information Processing theory, this study approaches nurses’ workplace gossip from the dynamic process of information perception, interpretation, and behavioral response, contextualized within the high-pressure, highly interactive, and emotionally demanding environment of the nursing profession, to systematically examine its motivations, consequences, and underlying mechanisms. At the motivational level, gossip among nurses is closely linked to their distinctive professional characteristics and individual needs. Compared with other professions, nursing work is characterized by frequent interpersonal interactions and communications, and nursing practice is deeply rooted in oral traditions, reflecting a highly interactive professional culture (9, 17, 43, 57). Moreover, the continuous, 24-h nature of patient care often constrains formal communication channels from fully addressing both clinical and emotional needs. Within this context, gossip functions as a critical supplementary communication mechanism (56). Besides, multiple studies indicate that nurses are exposed to particularly complex and challenging stressors (8, 9, 36, 37, 39, 43, 56). They routinely care for patients with poor health status and high stress levels, frequently encountering high-intensity situations such as emergency resuscitations and patient deaths. Consequently, nurses experience significant physical and emotional burdens. However, constrained by professional norms and societal expectations regarding nursing conduct, nurses are often required to work in environments where open emotional expression is restricted (17). In such contexts, gossip serves as a crucial outlet for emotional release and stress alleviation. Empirical evidence has shown that nurses frequently use gossip to express emotions such as anxiety and anger and to alleviate stress through informal conversations (8, 9, 17, 36, 37, 39, 43, 56, 57). Regarding associated consequences, it is noteworthy that the disruptive effects of gossip are magnified in nursing due to the high-risk nature of the profession. Harmful gossip (distracting nurses, eroding trust) can pose significant threats to patient safety. However, when properly managed, it can support career development and organizational growth. Understanding the mechanisms through which gossip functions within nursing teams is essential for developing effective management policies. Current research in nursing has not fully explored this aspect. Studies have found that workplace gossip exerts its influence through its interaction with perceptions of justice, emotional dynamics, and its impact on the organizational environment. Nevertheless, as a complex organizational phenomenon, workplace gossip in healthcare settings likely involves more intricate pathways and moderating factors, highlighting the need for comprehensive understanding.

### 4.1 Implications for nurse managers

The scoping review highlights the complex and distinctive role of workplace gossip in nursing. In fact, workplace gossip is an inherent and unavoidable aspect of organizational life. Even in well-managed institutions, it cannot be eliminated. Therefore, nursing managers should understand that the goal is not to eliminate gossip, but to provide reasonable guidance by recognizing its influencing factors, thereby maximizing its benefits and minimizing its negative impact.

Managers should recognize the value of positive workplace gossip and guide it to function as an informal learning tool, encouraging senior nurses to share their knowledge with newly hired nurses and fostering a supportive organizational environment. Also, managers can use workplace gossip as a means of providing social support to nursing staff. Informal recognition of nursing staff can serve as a valuable management tool to promote positive emotional experiences.

While harnessing the positive effects of gossip is important, it is equally crucial to minimize the occurrence and spread of negative gossip. Transparent communication strategies, such as anonymous feedback and regular meetings, are crucial to reduce gossip stemming from information gaps. Besides, existing evidence indicates a strong correlation between emotional stress and the occurrence of gossip (86, 87), emotional regulation and stress management training is also essential. Nursing managers can provide alternative outlets for emotional expression, such as stress-relief activities, to reduce transmission of negative workplace gossip. Moreover, organizational factors also play a role; environments with tenure diversity and self-management structures promote positive gossip, emphasizing the importance of cultivating supportive organizational conditions.

### 4.2 Prospects for future research

Although workplace gossip widely existed in healthcare institutions, current studies remain limited in several critical areas. The review firstly argue that it is necessary for scholars in nursing area to reach a consensus on definition of workplace gossip to provide a solid theoretical foundation for future studies. Then, existing studies pertaining on nursing workplace gossip is regionally limited, necessitating global evidence and cross-cultural studies to build a comprehensive theoretical framework. In addition, studies have primarily relied on survey questionnaires for data collection lacking reliable and validated measures of workplace gossip within the nursing context. Therefore, developing effective scales and models to enable accurate measurement of workplace gossip in nursing context will be an important research direction in future. The adoption of innovative methodologies, such as simulated dialog or topic mining of workplace gossip by machine learning models or AI algorithm may provide more significant and interesting insights for workplace gossip in nursing.

### 4.3 Limitations

Several limitations should be acknowledged in this current review. First, the available evidence is predominantly derived from English-language publications and is heavily concentrated within specific cultural contexts. While this distribution reflects the current landscape of scholarship on the topic, it inevitably constrains the cross-cultural transferability of the findings. Second, as this study is a scoping review, the primary aim was to provide a broad and integrative understanding of workplace gossip among nurses rather than to perform a strict quality-weighted synthesis. Therefore, no formal quality weighting was applied. Nevertheless, all included studies met the predefined inclusion and exclusion criteria, and their findings showed no substantial inconsistencies, which supports the overall reliability of the conclusions. Regardless of these limitations, the review still provides valuable insights for us to understand workplace gossip among nurses.

## 5 Conclusion

Grounded in Social Information Processing theory and situated within the unique occupational context of nursing, this study systematically examines the motivations, consequences, and underlying mechanisms of workplace gossip in nursing groups. The results highlight the prevalence of gossip in nursing settings and its potentially detrimental impact, emphasizing the need for evidence-based strategies to effectively guide workplace gossip. These findings provide initial, systematic insights into the dynamics of workplace gossip in nursing and offer evidence-based strategies for managers to guide such interactions effectively. Future research should focus on cross-cultural validation and intervention design to leverage gossip’s constructive potential, thereby maximizing its benefits in healthcare settings.
