# Correction: Motivations, consequences, and mechanisms of workplace gossip in nursing groups: a scoping review

**DOI:** 10.3389/fpubh.2026.1788753

**Published:** 2026-02-02

**Authors:** Frontiers Production Office

**Affiliations:** Frontiers Media SA, Lausanne, Switzerland

**Keywords:** workplace gossip, nurses, scoping review, motivations, consequences

Affiliation “Lucas College and Graduate School of Business, San José State University, San José, CA, United States” was erroneously given as “Department of Information Systems and Operations Management, College of Business Administration and Public Policy, California State University, Carson, CA, United States.”

The article type was erroneously given as Review. The correct article type is Systematic Review.

The original version of this article has been updated.

