# Supplementary material for: Motivations, consequences, and mechanisms of workplace gossip in nursing groups: a scoping review
Source: Front Public Health. 2025 Dec 18;13:1672645. doi: 10.3389/fpubh.2025.1672645 (PMC12756507; doi:10.3389/fpubh.2025.1672645)
Supplement: Supplementary file 1 [file Table_1.docx]

**Content**

**[Table 2](#_Toc9754)**

**[Table 1: Study characteristics 2](#_Toc27219)**

**[Search Strategy 14](#_Toc10905)**

**[The literature’s searching strategies used in PubMed (Results: 229) 14](#_Toc27496)**

| **Table****Table 1: Study characteristics** | | | | | | |
| --- | --- | --- | --- | --- | --- | --- |
| Author, year, country | Objective | Study design | Participants, locations | Main findings | Limitations | Citations |
| [Bulduk, et al. (2016)](#_ENREF_15)  Turkey | To determine nurses’ attitudes towards gossip and rumour. | Cross-section survey using The Gossip and Rumour Attitude Scale.  Descriptive characteristics survey using author designed questionnaire. | 268 nurses working in two private and two public hospitals. | 1. Female nurses, nurses working in private hospitals, those in administrative positions, and those employed in hospitals with clear job descriptions tended to have more positive perceptions of the effects of gossip.   (2) Nurses with a graduate degree and those with 1-3 years of work experience both tended to believe that gossip and rumours due to the specific reasons. | The study conclusions limited to Turkey, reflecting only the cultural context and professional nursing practices of the country. | 13 |
| [Ozluk and Ozcan (2021)](#_ENREF_70)  Turkey | To analyse nurses' attitudes toward and opinions on gossip and rumours. | Cross-section survey using The Gossip and Rumour Attitude Scale.  Descriptive characteristics survey using author designed written questionnaires. | 124 nurses working on the inpatient wards in a university hospital. | (1) Nurses identified gossip and rumours to be positive and did not consider them to be attributed to any reason.  (2) Nurses were negatively affected by gossip and rumours but showed passive reactions.  (3) Although nurses gossiped about personal and institution-related matters most, they also gossiped about patients and patient relatives. | The samples limited to nurses working on the inpatient wards and the location only in a university hospital of Turkey. | 2 |
| [Şantaş, et al. (2018)](#_ENREF_75)  Turkey | To determine the relationship between gossip functions, organizational revenge and job stress among working personnel in hospitals. | Cross-section survey using:  (1) Gossip Functions Questionnaire.  (2) 5-item scale developed by Wade. | 310 medical and administrative personnel, 61.9% of whom were nurses working in a university hospital. | (1) Relationship between gossip functions and job stress was not significant.  (2) Correlations between gossip functions and organizational revenge, and the correlation between job stress and organizational revenge are significant. | (1) Cross-section design of the study cannot infer causality.  (2) Other participants of study limited representativeness of the findings for the nurses.  (3) The sampling of this research is restricted only with the personnel in a city and a university hospital in Turkey. | 33 |
| **Table 1: Continued** | | | | | | |
| Author, year, country | Objective | Study design | Participants, locations | Main findings | Limitations | Citations |
| [Ceylan and Cetinkaya (2020)](#_ENREF_17)  Turkey | To determine the prevailing attitudes towards gossip and the patient privacy practices of nurses working in paediatric units. | Cross-section survey using The Gossip and Rumour Attitude Scale and Patient Privacy Scale.  Descriptive characteristics survey using author designed written questionnaires. | 112 nurses working in paediatric units included general paediatrics clinic, paediatric surgery clinic, paediatric emergency unit, child haematology clinic, neonatal intensive care unit, paediatric intensive care unit and outpatient treatment units in three hospitals (one university hospital and two public hospitals) | (1) Positive correlations were found between higher education level, education about patient privacy, reading patient rights regulations, and concern about patient privacy.  (2) Negative correlations were found between attitudes toward gossiping and the average scores on the patient confidentiality scale. | The samples limited to paediatric nurses and the location in province of western Turkey, both of which has limited the scope of the study. | 38 |
| [Georganta, et al. (2014)](#_ENREF_42)  European Countries | To examine the role of negative gossip in relation to burnout, job engagement, suboptimal care and patient safety in public hospitals. | Cross-section survey using:  (1) 7-itesms scale developed by Wittek and Wielers.  (2) The Positive and Negative Affect Schedule.  (3) The Maslach Burnout Inventory, the Utrecht Work Engagement Scale  (4) The Survey on Patient Safety Culture. | 532 health care professionals participated, 39.8% were nurses working in public hospitals of some European countries (Greece, Bulgaria, Romania, Turkey, Croatia and Republic of Macedonia). | (1) Negative gossip was positively related to emotional exhaustion and depersonalization through negative affectivity.  (2) Negative gossip was negatively correlated with patient safety and job engagement, while it was positively correlated with suboptimal care through burnout. | Other healthcare professionals limited representativeness of the findings for the nurses.  This study assessed negative gossip through a general measure of active participation in negative evaluative talk. | 134 |

| **Table 1: Continued** | | | | | | |
| --- | --- | --- | --- | --- | --- | --- |
| Author, year, country | Objective | Study design | Participants, locations | Main findings | Limitations | Citations |
| [Zoromba, et al. (2025)](#_ENREF_104)  Egypt | To unveil the mediating role of psychological detachment from work in the relationship between mistreatment by patients and nurses’ caring behaviours, as well as the moderating effect of supervisor positive gossip. | Cross-section survey using:  (1) The Supervisor Positive Gossip Scale.  (2) Recovery Experience Questionnaire.  (3) Four-item scale by Grandey et al.  (4) Caring behaviours inventory.  Descriptive characteristics survey using author designed questionnaires. | 341 nurses working in five hospitals across two cities.  (Of these five hospitals, one was a university hospital, two were government general hospitals, and two were health insurance hospitals). | 1. Mistreatment by patients was negatively related to nurses’ caring behaviours via an increased psychological detachment.   (2) Higher levels of supervisor positive gossip were shown to weaken the direct effect of mistreatment by patients on nurses’ psychological detachment from work and its indirect effect on their caring behaviours. | 1. Cross-section design of the study cannot infer causality. 2. It was conducted in two Egyptian cities, potentially limited the generalizability of the results.   (3) Self-reported data can lead to common method variance and issues of reliability. | 4 |
| [Durmus, et al. (2020)](#_ENREF_30)  Turkey | To reveal the relationship between nurse job performance and gossip level. | Cross-section survey using:  (1) Gossip Functions Questionnaire.  (2) Nurse Performance Scale.  Descriptive characteristics survey using author designed questionnaires. | 211 members, of which 91% were nurses while 9% were administrative, working in training and research hospitals or public hospitals. | (1) Gossip did not create a negative effect on nurse performance.  (2) Influence, one of determination characteristics of gossip, has negative relationship between nurses’ performance. | (1) Cross-section design of the study cannot infer causality.  (2) The sample size in this study is relatively small and limited to Turkey. | 15 |
| [Kim, et al. (2019)](#_ENREF_53)  South Korea | To identify the roles of perceived justice and insider status in relation to workplace gossip. | Cross-section survey using:  (1) 7-items scales developed by Wittek and Wielers.  (2) 20-items scale developed by Colquitt. (3) 6-items scale developed by Stamper and Masterson. | 329 nurses from four hospitals. | (1) Both procedural and distributive justice perceptions were positively associated with positive gossip behaviour toward the organization.  (2) Both interpersonal and informational justice perceptions were positively linked to positive gossip behaviour toward the supervisor and negatively related to negative gossip behaviour toward the supervisor.  (3) Distributive justice perceptions were negatively associated with negative gossip behaviour toward the organization. | (1) Cross-section design of the study cannot infer causality. (2) The use of a single rater is problematic owing to concerns about common method bias.  (3) The study focused only on individual-level factors like justice, perceived insider status, and gossip. However, motivation for workplace gossip among nurses may also stem from organizational and interpersonal factors. | 91 |
| **Table 1: Continued** | | | | | | |
| Author, year, country | Objective | Study design | Participants, locations | Main findings | Limitations | Citations |
| [Elsayed (2024)](#_ENREF_32)  Egypt | To assess the nurse managers' management of grapevine communication among nursing staff. | Cross-section survey using:   1. Grapevine Communication   Structured Questionnaire   1. Nurse Managers’ Management of Grapevine Communication Questionnaire. | 35 nurse managers and 260 staff nurses from Tanta  University Main Hospital. | (1) Nurse managers saw grapevine communication as unchecked information sharing, while most nurses believed that grapevine communication widens the gap between senior management and nursing staff.  (2) Most nurse managers had an elevated level of management in creating trust relationships with the nursing staff.  (3) The causes of grapevine communication were correlated with its management, except in maintaining open communication channels and managing informal groups. | (1) Cross-section design of the study cannot infer causality. | 0 |
| Waddington (2005)  UK | To explore the role of gossip in the expression and management of emotion in nursing work. | In-depth qualitative interview. | 10 Clinical Nurse Specialists in NHS (National Health Service) Hospital Trust. | Gossip was a feature of nurses’ emotional labor in that it provides the opportunity for the expression of authentic feelings about patients and colleagues, which cannot be expressed in public. | The methodology relied upon participants’ retrospective recollection of their experience of gossip, accuracy is inadequate. | 67 |
| **Table 1: Continued** | | | | | | |
| Author, year, country | Objective | Study design | Participants, locations | Main findings | Limitations | Citations |
| [Kim, et al. (2021)](#_ENREF_54)  South Korea | To examine how group diversity affects individual group members’ negative gossip about their colleagues and how this linkage is altered by group structure. | A longitudinal design with a two-wave survey (Time 1 and Time 2):  (1) At Time 1, information was collected using questionnaires designed by the authors.  (2) At Time 2, measurement was conducted using a 7-item scale developed by Wittek and Wielers. | 312 nurses employed in 39 nursing groups from four healthcare institutions. | Employees in groups with high tenure diversity were less likely to negatively gossip about their coworkers when the group had a greater self-managing structure. | (1) Sample was limited to South Korean workers.  (2) While examining group tenure diversity, other group member attributes may also influence the nature of gossip in organizations.  (3) Inter-rater reliability may be a concern, as information on self-managing structures was provided only by a senior nursing manager. | 14 |
| [Altuntaş (2017)](#_ENREF_4)  Turkey | To determine whether the gossip is used in organizational communication by academicians. | A qualitative method using a questionnaire designed by the authors, which focused on gossip between academicians. | 177 participants, 31.1% in the Faculty of Health Sciences Nursing Department from a public university. | Academicians in the field of health occasionally gossiped, mainly about their working conditions. They did so to share information face-to-face with their friends. When they felt angry, remorseful, or stressed, they tried not to gossip again. However, after gossiping, their stress levels decreased, their self-confidence increased, and they felt relieved. | (1) Sample was restricted only with the academicians in a city and public university in Turkey.  (2) Other participants of study limited representativeness of the findings for the nurses. | 3 |
| [Altuntaş, et al. (2014)](#_ENREF_3)  Turkey | To determine how nurses use gossip as an informal communication channel in organizations. | A qualitative method using a questionnaire designed by the authors was employed to test a descriptive model proposed in the study. | 264 nurses from four hospitals, 61.7% of whom work in internal medicine or surgery clinics. | (1) Nurses used gossip as an informal communication style in their institutions.  (2) Nurses gossiped with their friends to share information face-to-face when they feel angry and gossip about matters related to working conditions. | 1. The findings were limited to nurses employed in four hospitals in a province located in eastern Turkey.   (2) The inadequate number of studies of the topic in nursing limited the discussion of the study’s findings. | 50 |

| **Table 1: Continued** | | | | | | |
| --- | --- | --- | --- | --- | --- | --- |
| Author, year, country | Objective | Study design | Participants, locations | Main findings | Limitations | Citations |
| [Begemann, et al. (2021)](#_ENREF_12)  Germany | To explore the dynamic nature and social embeddedness of workplace gossip. | Qualitative method and data captured with built-in microphone. | 62 nurses from eight nursing teams in four different elderly care homes. | Nearly half of the observed gossip events were neutral (45.7%), followed by negative (27.2%), ambiguous (20.1%), and positive gossip (7%)  The main functions of gossip with different valences were different. | (1) The sample size in this study is relatively small.  (2) The research context of virtual meetings overlooks some of the social functions of informal communication.  (3) The study cannot rule out social desirability bias in the observed meeting behaviour. | 10 |
| [Aghbolagh, et al. (2021)](#_ENREF_1)  Iran | To analyse gossip in an organizational context and to show its differences with gossip in the non-organizational environments. | Qualitative method using semi structured interview conducted around topic of "What do employees say about each other?". | 9 nurses from a teaching hospital.  9 nurse supervisors.  9 nurse managers. | The topics of gossip in a hospital can be divided into eight main categories, and 34 sub-categories all identifiable by special topics.  The gossip topics at different levels among nurses, administrators, and managers had significant differences. | (1) The sample size in this study is relatively small.  (2) Due to the COVID-19 pandemic, hospital employees faced high workload and stress, so the results may differ under normal conditions. | 10 |
| [Waddington and Fletcher (2005)](#_ENREF_96)  UK | To examine the relationship between gossip and emotion in health-care organizations. | Mixed methods included repertory grid technique, in-depth interviews and structured diary records. | 96 qualified nurses included newly qualified nurses, nurse managers, senior nurses, and nurses with specialist roles. | Gossip was used to express a range of emotions including care and concern about others, anger, annoyance and anxiety, with emotional outcomes that include feeling reassured and supported. | The study does not provide a universally accepted and precise definition of gossip. | 157 |
| **Table 1: Continued** | | | | | | |
| Author, year, country | Objective | Study design | Participants, locations | Main findings | Limitations | Citations |
| [Waddington (2005)](#_ENREF_95)  UK | To explore the characteristics and function of gossip in nursing and health care organizations. | Mixed methods combined qualitative (such as diary study) and quantitative (such as data analysis) | 96 nurses  Phase 1: 66 qualified nurses working in varied organizational settings and roles.  Phase 2: 10 specialist nurses working in one NHS Trust.  Phase 3: 20 qualified nurses and midwives working in varied organizational settings and roles. | Nurses used gossip as a means of expressing and communicating emotions, but there was a danger that the immediacy of gossip as a 'quick fix' solution might mask the need to adopt different methods of managing the situation at the individual, team, or organizational level. | (1) Self-reported data can lead to common method variance and issues of reliability.  (2) Data missingness may occur during daily diary studies. | 132 |
| [Out (2005)](#_ENREF_68)  Canada | To explore the process of self-labelling and how women, and nurses in particular, come to attach meaning and significance to bullying that they may experience in the workplace. | Mixed methods combined qualitative and quantitative. | 385 nurses working in the College of Nurses of Ontario. | (1) Being maliciously gossiped about is a manifestation of being bullied. Verbal abuse was found to be more strongly associated with self-labelling than with behaviours.  Nurses in the bullied, self-labelling group were hypothesized to report lower levels of job satisfaction, higher levels of burnout, and greater psychological distress than nurses in the bullied, non-labelling group. | (1) Cross-section design of the study cannot infer causality.  (2) The Negative Acts Questionnaire (NAQ) used in this study does not provide a comprehensive assessment of the bullying behaviours that nurses may experience. | 21 |
| [Babaei Aghbolagh (2016)](#_ENREF_7)  Unspecified country | To review the social functions of gossip especially in the hospitals among nurses. | Review: from the aspects of different functions of gossip. |  | Gossip had positive and negative results at both the individual and organizational levels.  Nurses can reduce their stress and anxiety by WG. Meanwhile, uncontrolled or negative gossip can be harmful for organizational interests. | (1) The study did not deeply analyse causes, transmission mechanisms of gossip.  (2) The study did not address how individual differences affect gossip behaviour and its impact among nurses. | 19 |

| **Table 1: Continued** | | | | | | |
| --- | --- | --- | --- | --- | --- | --- |
| Author, year, country | Objective | Study design | Participants, locations | Main findings | Limitations | Citations |
| [Chase and Stuart (1995)](#_ENREF_19)  Unspecified country | To explore the role of Psychiatric CNS in rumour management in the nursing system. | Phenomenological: provided examples to give the strategies and approaches for managing rumour and gossip in nursing content. |  | Unauthorized information, known as rumours or gossip, can be a powerful force within a nursing system and may influence the means of accessing and managing it. | (1) The study was too old to reflect recent advances in rumour management and psychiatric nursing.  (2) The proposed interventions do not fully account for variations in hospital culture, management styles, and nurse differences, limiting practical application. | 7 |
| [Dowd, et al. (1997)](#_ENREF_29)  Unspecified country | To explore the impact and management measures of rumours in the healthcare system. | Review: from the aspects of detecting, managing, and consequences of rumour and gossip. |  | Rumour and gossip may be negative or positive, and health care supervisors should monitor the grapevine and consider themselves personally responsible for transmitting accurate information whenever possible to ensure that rumour and gossip do not have a negative effect on the department or institution. | The study relied mainly on theoretical discussions with no large-scale surveys or experiments that limited reliability and generalizability of its conclusions. Besides, it did not deeply explore the psychological and sociological factors that impact the spread of rumours and gossip. | 14 |

| **Table 1: Continued** | | | | | | |
| --- | --- | --- | --- | --- | --- | --- |
| Author, year, country | Objective | Study design | Participants, locations | Main findings | Limitations | Citations |
| [Prestia (2021)](#_ENREF_73)  Unspecified country | To describe the existence of informal networks, their organizational influence, varying informal communication patterns, and to provide nurse leaders with the knowledge to recognize and utilize these networks. | Review: from informal communication perspective. |  | (1) Nurse leaders should recognize, minimize, and coexist with the informal communications network within their organization.  (2) Employees deserve timely and accurate information from their leaders to avoid the creation of damaging alternative truths. | The study relied mainly on theoretical discussions with no large-scale surveys or experiments that limited reliability and generalizability of its conclusions. | 5 |
| [Ribeiro and Blakeley (1995)](#_ENREF_74)  Unspecified country | To present an analysis of gossip and rumour and discuss strategies for their prevention and control. | Review: the determinants, mediating factors, and consequences of rumour and gossip. |  | (1) Rumours and gossip were prevalent and common in the workplace.  (2) The spread of rumours and gossip tended to increase during times of organizational turbulence, uncertainty, and change.  (3) Rumours and gossip serve multiple social functions, having both negative impacts and positive roles. Managers should actively explore strategies for managing gossip. | The study relied mainly on theoretical discussions with no large-scale surveys or experiments that limited reliability and generalizability of its conclusions. Besides, it did not account for dynamic impact of internal and external environmental. | 46 |
| **Table 1: Continued** | | | | | | |
| Author, year, country | Objective | Study design | Participants, locations | Main findings | Limitations | Citations |
| [Baltimore (2006)](#_ENREF_9)  Unspecified country | To explore the challenges faced by newly hired nurses and the impact of experienced nurses on them. | Phenomenological: discussed the gossip from perspective of organization culture, especially focused on nurse-to-nurse behaviour. | . | (1) New nurses were particularly vulnerable to being the subject of gossip or badmouthing simply due to their unfamiliarity with a new setting.  (2) Managers who actively intervened in gossip problems increased productivity and trust, while improving relationships and reducing tension. | The study relied mainly on personal experience, observations, and literature, without empirical data to support. | 149 |
| [Waddington (2016)](#_ENREF_94)  Unspecified country | To argue that gossip is a neglected aspect of organizational communication and knowledge, and an under-used management resource. | Critical analysis: provided viewpoint that gossip provides opportunity for rational knowledge about systemic failure and poor practice in healthcare to surface. |  | Gossip can be a valuable early warning indicator of risk and failure in healthcare systems and a reflection of "the-problem-behind-the-problem". As a result, whether and how to engage with gossip is an ethical decision that should be considered in decision-making processes. | The study relied mainly on personal experience, observations, and literature, without empirical data to support. | 26 |
| [Thomas and Rozell (2007)](#_ENREF_87)  Unspecified country | To highlight the positive and negative aspects of gossip and provides strategies to help nursing professionals effectively manage this workplace issue. | Critical analysis: provided viewpoint that nursing manager need to find way to control the type and level of gossip that exist in their units. |  | Nurse managers need to identify and manage the types and levels of gossip, leveraging its positive aspects while mitigating its negative effects. | The study relied mainly on personal experience, observations, and literature, without empirical data to support. | 56 |
| **Table 1: Continued** | | | | | | |
| Author, year, country | Objective | Study design | Participants, locations | Main findings | Limitations | Citations |
| [Laing (1993)](#_ENREF_56)  Unspecified country | To scrutinize gossip from historical, analytical and feminist perspectives to explore how the functions of gossip may contribute to the socialization of nurses to their professional role and to their work culture. | Review: from three primary functions of gossip, included information, influence, and entertainment. |  | 1. Gossip provided information, influence, and insight regarding acceptable nursing practices and was one way for novices or new employees to become socialized into their professional and work roles.   (2) Gossip was often viewed as a communication style unique to women, but both men and women engage in gossip on similar topics, with women typically spending more time on it. | The study relied mainly on personal experience, observations, and literature, without empirical data to support. | 76 |
| [Montgomery (2024)](#_ENREF_65)  Unspecified country | To explore the importance of informal communication in adverse events and to understand the background of workplace gossip and its role in the healthcare system. | Critical analysis: provided viewpoint that the functions of gossip may contribute to socialization of nurses to their professional role and work culture. |  | (1) Gossip can allow staff to detect errors, reveal safety issues, and expose unethical behaviour.   1. Gossip can fulfil the self-determination theory’s main elements, by making individuals feel more competent, in control, and more socially related to their colleagues. | The study relied mainly on personal experience, observations, and literature, without empirical data to support. Besides, the analysis focused on healthcare cases from countries like the UK, without fully considering differences in healthcare systems across other countries and regions. | 1 |
| **Table 1: Continued** | | | | | | |
| Author, year, country | Objective | Study design | Participants, locations | Main findings | Limitations | Citations |
| [Fowler (2019)](#_ENREF_41)  Unspecified country |  | Phenomenological: a simple analysis and discussion that focus on how to be a good nurse in a moral sense. |  | Guidance for being a good nurse can come from gossip. | (1) The study relied mainly on personal experience, observations, and literature, without empirical data to support.  (2) The discussion of concept of 'gossip' focused mainly on its historical meaning and representation in nursing literature, without exploring its diverse forms, transmission methods, and broader impact in contemporary nursing practice. | 5 |
| [Scott (2003)](#_ENREF_77)  Unspecified country |  | Phenomenological: explored the gossip habits of nurses. |  | Although gossip was prevalent among nursing staff and could sometimes have negative effects, considering its positive role in helping nurses cope with work-related stress, it can be viewed as a 'necessary evil' within the healthcare system. | (1) The study relied mainly on personal experience, observations, and literature, without empirical data to support.  (2) The study did not explore strategies and methods for effectively reducing negative gossip and improving the nursing work environment. | 4 |

# **Search Strategy**

| **The literature’s searching strategies used in PubMed (Results: 229)** | | |
| --- | --- | --- |
| **No** | **Searches** | **Results** |
| #1 | "Nurses"[Mesh] | 102565 |
| #2 | Nurs*[Title/Abstract] | 577110 |
| #3 | #1 OR #2 | 611657 |
| #4 | gossip [Title/Abstract] | 565 |
| #5 | whispers [Title/Abstract] | 119 |
| #6 | rumors [Title/Abstract] | 937 |
| #7 | grapevine [Title/Abstract] | 6123 |
| #8 | "Informal communication"[Title/Abstract] | 150 |
| #9 | "Informal knowledge transfer"[Title/Abstract] | 4 |
| #10 | "Informal knowledge sharing"[Title/Abstract] | 9 |
| #11 | "Individual behaviour"[Title/Abstract] | 765 |
| #12 | #4 OR #5 OR #6 OR #7 OR #8 OR #9 OR #10 OR #11 OR #12 | 8642 |
| #13 | #3 AND #12 | **229** |
